# FDM data driven U-Net as a 2D Laplace PINN solver

**DOI:** 10.1038/s41598-023-35531-8

**Published:** 2023-06-05

**Authors:** Anto Nivin Maria Antony, Narendra Narisetti, Evgeny Gladilin

**Affiliations:** Leibniz Institute of Plant Genetics and Crop Plant Research, OT Gatersleben, Corrensstr. 3, 06466 Seeland, Germany

**Keywords:** Computational biophysics, Computational models, Computer science, Computational science, Applied mathematics, Information technology, Software

## Abstract

Efficient solution of partial differential equations (PDEs) of physical laws is of interest for manifold applications in computer science and image analysis. However, conventional domain discretization techniques for numerical solving PDEs such as Finite Difference (FDM), Finite Element (FEM) methods are unsuitable for real-time applications and are also quite laborious in adaptation to new applications, especially for non-experts in numerical mathematics and computational modeling. More recently, alternative approaches to solving PDEs using the so-called Physically Informed Neural Networks (PINNs) received increasing attention because of their straightforward application to new data and potentially more efficient performance. In this work, we present a novel data-driven approach to solve 2D Laplace PDE with arbitrary boundary conditions using deep learning models trained on a large set of reference FDM solutions. Our experimental results show that both forward and inverse 2D Laplace problems can efficiently be solved using the proposed PINN approach with nearly real-time performance and average accuracy of 94% for different types of boundary value problems compared to FDM. In summary, our deep learning based PINN PDE solver provides an efficient tool with various applications in image analysis and computational simulation of image-based physical boundary value problems.

## Introduction

Rapid advances in biomedical imaging lead to the generation of ever-expanding amounts of image data. In many applications, image analysis is mainly restricted to the derivation of relatively simple quantitative descriptors of targeted structures such as color, volume, area, and shape. However, image series can also provide deeper insights into underlying physical properties and behavior that stand behind dynamic changes of optically monitored biological structures^1–3^.

In general, consistent physics-based modeling requires a numerical solution of a boundary value problem (BVP) which is given by the governing partial differential equation (PDE) or constitutive law (e.g., equations of continuum mechanics, fluid dynamics, diffusion) and prescribed boundary conditions. For this task, conventional domain discretization techniques such as Finite Difference (FDM)^4^, Finite Element (FEM)^5^, Boundary Element (BEM)^6^, and mesh-free methods^7^ were frequently used in the context of biomedical applications^8,9^. Conventional numerical techniques are, however, not suitable for real-time applications and also require advanced skills for adaptation to new data and research goals. To reduce the computational demand on conventional numerical solvers, several approaches have been studied including surrogate^10,11^, model order reduction^12–17^, or multigrid techniques^18–20^. Although these advanced methods are capable of reducing computational costs, they do not capture the entire spectrum of computational tasks including real-time, inverse, and/or non-linear problems that are not yet satisfactorily addressed in many interdisciplinary and, in particular, biomedical applications. In recent years, alternative approaches to solving physics and image-based BVPs using data-driven neural network models enjoyed increasing popularity. The so-called Physically Informed Neural Networks (PINNs)^21^ trained on a large amount of representative data learn to infer complex physical relationships directly from data. With a sufficient amount of available data, PINNs can establish a mapping between the input and the output data (e.g., source and target images) without embedding the physical laws directly into neural networks. Being capable of overcoming one of the major technical burdens of numerical modeling, a laborious and error-prone discretization of complex spatial-temporal domains, PINNs hold a promise to bridge a gap between large data and sophisticated mechanism-based modeling with nearly real-time performance. Moreover, the spectrum of applicability of PINNs covers not only forward but also even more computationally challenging inverse problems^21–26^. In recent years, many approaches to the data-driven approximation of physical mechanisms using deep neural networks were reported^18,19,27–30^, and works^19,30^ investigate the problem using CNNs. Convolutional Neural Networks (CNNs)^31^ are known to show superior performance compared to conventional methods and sparse neural network techniques, especially by application to computer vision problems that require higher-order cognitive abilities. The Deep Learning coding platforms like Tensorflow, PyTorch, and Keras are meanwhile widely used among the AI community.

However, most known PINN frameworks are mostly available with special software platforms DeepXDE^32^, Nvidia SimNet^33^, NeuroDiffEq^34^, which lacks flexible definition and solution of physical BVPs on arbitrary image domains. The GPU-based numerical simulation platforms based on conventional approaches like NiftySim^35^, and SoFa^36^ are applications specific that requires different implementations for different problems.

In this work, we aim to investigate the capability of deep learning methods to effectively solve an image and physics-based BVP with arbitrary boundary conditions. In particular, here we rely on the basic network architecture of U-Net, which was originally developed for a broad range of image segmentation problems^37^. In contrast to image segmentation problems, solving physical BVPs implies multi-class or, more general, optimization problems. Consequently, two modifications of the U-Net with multi-class and custom loss functions were introduced and trained on a large set of reference FDM solutions of the 2D Laplace PDE.

Our manuscript presents a theoretical and experimental framework of this is structured as follows. First, we describe the numerical FDM scheme which was used for solving 2D Laplace problems and generation of a large set of reference images for subsequent training of deep learning models. Then, the results of the PINN model performance by application to forward and inverse 2D Laplace problems are presented and compared with the reference FDM solutions. Our experimental results show that our PINN models are capable of resembling conventional numerical solutions for new unseen data with remarkable accuracy. Finally, present limitations and further improvements of our PINN approaches are discussed.

## Methods

### 2D Laplace PDE and BVP

In this work, we focus on the solution of the 2D Laplace partial differential equation (PDE), which arises in mathematical physics through the description of problems of heat propagation and diffusion. The 2D Laplace PDE belongs to the category of elliptic PDEs of the second order:

1 $$\begin{aligned} \Delta u = \frac{\partial ^ 2 u}{\partial x^2} + \frac{\partial ^ 2 u}{\partial y^2} = 0, \end{aligned}$$

where $u=u(x,y)$ is a scalar variable defined on a 2D spatial domain, and, thus, in general, a function of coordinate. Non-trivial solutions of Eq. (1) with the zero right-hand sides exist only if non-zero values of $u = u_{D} \ne 0$ are defined on some parts of the spatial domain $\Omega _D \in \Omega$. Often, the prescribed values of *u* are defined on some external or internal boundaries $\Gamma \subset \Omega$. Such boundary conditions are known as Dirichlet boundary conditions. The problem of finding a solution to the PDE for the given boundary conditions is termed the Boundary Value Problem (BVP).

### Fundamental solution

Analytical solutions of Eq. (1) are known only for some spatial cases of particularly simple (symmetric) boundary conditions. One such special case is a solution of the inhomogeneous Laplace PDE with the right-hand side in form of the Dirac point function:

2 $$\begin{aligned} \Delta u(r) = -\delta (r-r'), \end{aligned}$$

where $r(x,y)-r'(x',y')$ is a vector pointing from the coordinate $(x',y')$, where the Dirac impulse was applied, to some other coordinate (*x*, *y*) of the infinite 2D domain $\Omega$. The solution of Eq. (2) also known as the fundamental solution of 2D Laplace PDE is given by^38^

3 $$\begin{aligned} u(r) = -\frac{1}{2\pi }ln\mid r-r'\mid . \end{aligned}$$

### Numerical solution using the finite difference method

The BVP defined by Eq. (1) with arbitrary boundary conditions can, in general, be solved only numerically.

In this work, the Finite Different Method on the regular 2D image grid is applied for this purpose. In particular, FDM approximates the derivatives by differences between the variable values between neighbor grid nodes, i.e.

4 $$\begin{aligned} \frac{du}{dx} = \frac{u_{i+1}-u_i}{x_{i+1}-x_i} . \end{aligned}$$

Accordingly, the FDM equation of the second-order 2D Laplace PDE on the regular grid of image nodes (i.e. pixels) takes the form:

5 $$\begin{aligned} ( \Delta u)_{ij} = \frac{u_{i+1,j} + u_{i-1,j} -2u_{i,j}}{ \Delta x^2} + \frac{u_{i,j+1} + u_{i,j-1} -2u_{i,j}}{ \Delta y^2} = 0, \end{aligned}$$

where (*i*, *j*) are indices of image pixels in the Euclidean system coordinate (XY). Consideration of Eq. (5) for all image pixels leads to a linear system of equations for unknown nodal values $u_{ij}$, which after implementation of known nodal values (i.e. prescribed Dirichlet boundary conditions) can be compactly written in a matrix form as follows:

6 $$\begin{aligned} A\,u = b, \end{aligned}$$

where *A* is a symmetric and positive definite matrix and *b* is the right-hand side vector resulting from the implementation of known values of *u*.

In this work, the FDM discretization of Eq. (5), its assembly into Eq. (6) followed by a subsequent numerical solution using the Preconditioned Conjugate Gradient (PCG) method was implemented under MATLAB 2021a. The FDM solver implemented as described above was firstly evaluated by a direct comparison vs. the exact analytic solution of the 2D Laplace PDE [i.e. the fundamental solution in Eq. (3)] and then applied for solving of BVPs arbitrary boundary conditions.

### Generation of a reference set of FDM solutions

To appropriately train the PINN model to accurately emulate solutions of the 2D Laplace PDE for arbitrary boundary conditions, a large set of pairwise reference BVP images and their FDM solutions has to be generated. For this purpose, a set of BVP images with different geometrical patterns of Dirichlet domains and distributions of prescribed values on these subdomains was generated. For the definition of different geometrical patterns different shape primitives such as blobs, points, lines, triangles, rectangles, circles, etc., and their random variations and perturbations of scale and location were used. In the next step, prescribed values of $u\in [25,255]$ were assigned to pixels of each geometrical pattern. By constructing the distribution of prescribed values several perturbation strategies were used. First, strategy is based on the generation of constant, gradient, or random values. The next factor influencing the distribution was the randomization of the parameters of these distributions. For example, in the case of gradient patterns, the direction and the magnitude of the gradient were randomized within a range of some admissible bounds.

### U-Net as PINN PDE solver

Our approach to emulation of 2D Laplace PDE using a PINN model is based on the adaptation of the U-Net image segmentation framework. However, in contrast to typical image segmentation tasks solving continuous physical BVPs requires the introduction of suitable loss functions. Here, we use and investigate the performance of U-Net with two alternative loss functions including (i) multi-class Sparse Categorical Entropy (SCE) (Eq. 7) and (ii) the Mean Squared Error (MSE) (Eq. 8):

7 $$\begin{aligned} L_{\text {SCE}} = -\frac{1}{N} \sum _{i=1}^N \sum _{j=1}^M z_{i,j} \log (p_{i,j}), \end{aligned}$$

where $z_{i,j}$ indicates whether the *i*-th pixel is classified correctly for the given class label *j*, $p_{i,j}$ is the confidence score of the classification, *N* is the number of pixels and *M* is the number of classes, and

8 $$\begin{aligned} L_{\text{MSE}} = \operatorname*{arg\,min}_\theta \frac{1}{N} \sum_{i = 1}^{N} ((q_i;\theta) - q_i^* )^2, \end{aligned}$$

where $q_i^*$ is the ground truth value on the *i*-th image pixel , $q_i$ is the predicted value for the given network parameter $\theta$ and *N* is the number of image pixels. Accordingly, these two U-Net modifications are further termed MC U-Net and MSE U-Net.

In addition, the entire U-Net architecture has to be modified to achieve good performance by solving physical BVPs. The modifications of the original U-Net are as follows. The dropout layers have been replaced with batch normalization layers in our study. Batch normalization standardizes the input for every mini-batch which increases the stability and the speed of the training of models. Without batch normalization, When there is a change in the input distribution to the model, the hidden layers try to adapt to the new distribution thus causing an internal covariate shift^39^. The modifications of the U-Net architecture introduced in this work are summarized in Table 1.

**Table 1:** Comparison of the PINN solver of the 2D Laplace PDE vs. original U-Net.

Parameters	PINN solver	Orig. U-Net
Contracting path kernels	7 $\times$ 7	3 $\times$ 3
Expansive path kernels	3 $\times$ 3	2 $\times$ 2
Stride	2 $\times$ 2	1 $\times$ 1
Padding	Unpadded	Padded with zeros
Depth	4	4

#### Forward PINN models

Forward models are trained to predict the FDM solutions of BVPs given by the 2D Laplace PDE with prescribed boundary conditions. For training forward models, input data were defined by synthetic BVP images generated as described above, while output data is given by FDM solution of BVP images. Consequently, the forward models were trained to correctly assign the intensity values of image pixels to one of a total of 256 possible classes (i.e. intensity values) of 8-bit images. Such a model is purely data-driven and does not explicitly include physical equations as a part of cost functions.

#### Inverse PINN models

Inverse models are trained by reversing input and output datasets that were used for forward model training. Consequently, the inverse models aimed to learn how to reconstruct the original BVP image from its (FDM) solution.

### Training models

Both forward and inverse PINN models were developed under Python 3.8 using TensorFlow^40^ with Keras API. Furthermore, some image processing operations such as reading and training data preparation were done using PIL, NumPy^41^, and Scikit-image^42^. Followed by, PINN models were trained on a GPU machine with a Linux operating system (Intel(R) Core (TM) i7-10700K CPU @3.80 GHZ) and NVIDIA RTX 3090-24GB graphic card. Regarding model training configuration, prepared datasets were partitioned into training and validation in the ratio of 85:15 respectively based on our experiences and previous studies^43,44^. The initial weights of the PINN model were defined randomly with zero mean and standard deviation of 0.05, as proposed by^45^. Here, Adam optimizer^46^ is used to optimize the model and to improve the performance on training datasets. Then MC U-Net models were trained for 500 epochs with 24 convolutional channel features with a learning rate of 0.0001 and a batch size of 256 and the MSE U-Net models were trained for 2000 epochs with 24 convolutional channel features with a learning rate of 0.0001 and a batch size of 128.

### Validation of model predictions

The predictions of forward and inverse PINN models ($I_{\text {PINN}}$) are validated by direct comparison with reference FDM solutions ($I_{\text {FDM}}$). To account for admissible differences due to rounding of floating point values to integer-valued image intensities, differences of up to one intensity value (i.e. deviations of plus-minus one segmentation class) were tolerated, i.e.

9 $$\begin{aligned} D = \mid I_{\text {PINN}} - I_{\text {FDM}} \mid = {\left\{ \begin{array}{ll} 1,&{} \text {if } D\le 1\\ 0, &{} \text {otherwise}. \end{array}\right. } \end{aligned}$$

The performance of PINN models vs. FDM solutions was evaluated using conventional derivatives of the multi-class confusion matrix, which in our case counts 256 classes (i.e. intensity values of an 8-bit image). The same metrics have been used to evaluate the MSE U-Net models as the results are 8-bit images and the pixel values rounded to the nearest integer value. In particular, accuracy and F1 score were assessed for every test image:

10 $$\begin{aligned}{} & {} Accuracy = \frac{TP+TN}{TP + FN + FP + TN}, \end{aligned}$$

11 $$\begin{aligned}{} & {} F1 = \frac{2*(Precision * Sensitivity)}{Precision + Sensitivity}, \end{aligned}$$

where Precision and Sensitivity are

12 $$\begin{aligned}{} & {} Precision = \frac{TP}{TP + FP}, \end{aligned}$$

13 $$\begin{aligned}{} & {} Sensitivity = \frac{TP}{TP + FN}. \end{aligned}$$

In addition, the Mean Absolute Error (MAE) and Mean Squared Error (MSE) of all image pixels were computed:

14 $$\begin{aligned}{} & {} MAE = \frac{1}{N}\sum _{i=1}^{N}|p_i - p_i^*| \end{aligned}$$

15 $$\begin{aligned}{} & {} MSE = \frac{1}{N}\sum _{i=1}^{N}(p_i - p_i^*)^2 \end{aligned}$$

where $p_i^*$ is the ground truth value on *i*-th image pixel and $p_i$ is the prediction value. Particularly large outliers of the PINN predictions were localized by thresholding the difference between the FDM and PINN solutions:

16 $$\begin{aligned} Outlier = {\left\{ \begin{array}{*{20}l} \text{yes}, &{} \text {if } D\ge tsh, &{} tsh = 2\\ \text {no}, &{} \text {otherwise}. \end{array}\right. } \end{aligned}$$

## Results

### Analytical and numerical solutions of the 2D Laplace PDE

**Figure 1:**
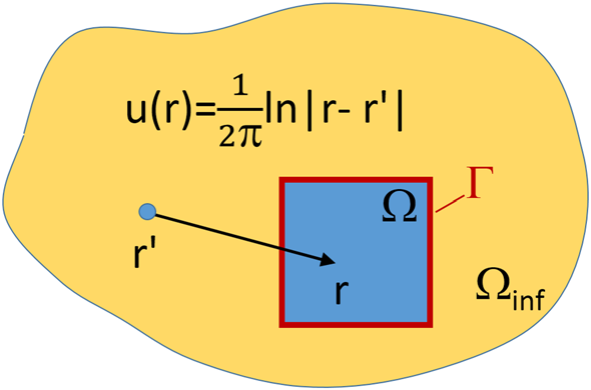
Validation of FDM solution vs. analytical (fundamental) solution of the 2D Laplace PDE *u*(*r*(*x*, *y*)) is performed for the quadratic subdomain $r(x,y)\in \Omega$ of the infinite medium $\Omega _{\text {inf}}$. For validation, only inner points (i.e. inner image pixels) $r(x,y)\in \Omega \backslash \Gamma$ were considered, while the boundary values were set equal to the fundamental solution $u(r(x,y))=u_{\text {fs}},~r(x,y)\in \Gamma$ and used as boundary conditions for computing the FDM solution on inner domain nodes.

The reference set of numerical solutions of the 2D Laplace PDE was generated using the Finite Difference Method as described in the Methods section. To validate the accuracy of the FDM solver, numerical solutions were compared vs. the fundamental solution of the 2D Laplace PDE, see Eq. (3). For this purpose, analytical and numerical solutions were computed for a quadratic subdomain $\Omega$ of the infinite medium $\Omega _{\text {inf}}$ which does not include the source point ($|r-r'|>0\,,\forall r(x,y) \in \Omega$), where the fundamental solution exhibits a singular behavior ($u(r=r') \rightarrow \infty$), see Fig. 1. The validation of the FDM solution vs. the fundamental solution of the 2D Laplace PDE was performed for the inner points of the subdomain $r(x,y)\in \Omega \backslash \Gamma$ only. The boundary values of the fundamental solution $u(r(x,y)),\,r(x,y)\in \Gamma$ we used as prescribed boundary conditions for subsequent computation of the FDM solution inside $r(x,y)\in \Omega \backslash \Gamma$. The theoretical basis for this approach is given by the Gaussian integral theorem, which states that the solution inside of a closed spatial domain is uniquely defined by its boundary values.

**Figure 2:**
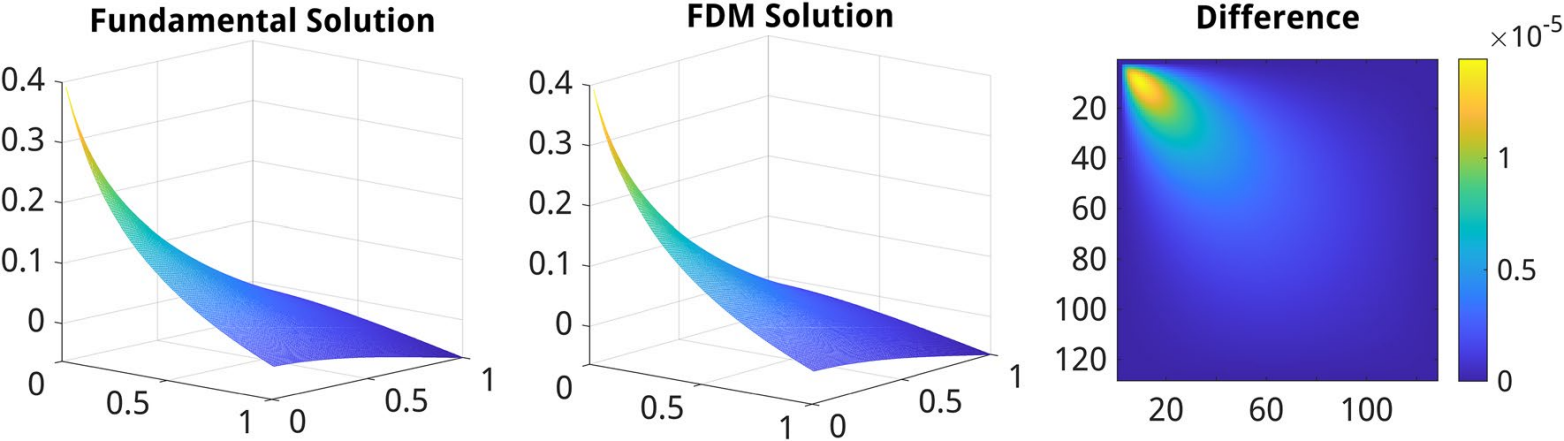
Comparison of the fundamental solution of the 2D Laplace PDE vs. a numerical solution computed using the FDM for a 128 $\times$ 128 image subdomain. From left to right: plot of the fundamental solution (FS) for a 128 $\times$ 128 sampled subdomain of the infinite medium, plot of the FDM solution computed from the FS values defined on the boundary of the 128 $\times$ 128 image, the difference between the fundamental and FDM solutions.

Figure 2 shows a comparison of the analytical solution vs. FDM calculated on the 128 $\times$ 128 image grid of the subdomain $\Omega$. As one can see, the absolute difference between the fundamental and FDM solutions amounts up to $1\text {e-}5$ reaching the maximum in the vicinity of the singular source point.

### Generation of the reference set of BVP images and their FDM solutions

**Figure 3:**
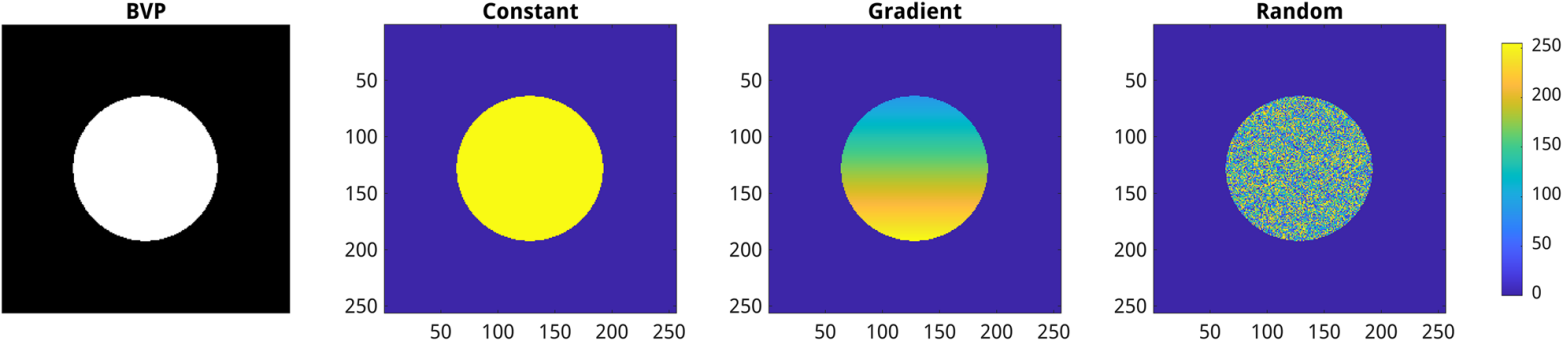
Example of BVP image generation. From left to right: binary image (i.e. geometrical pattern) for the definition of prescribed (Dirichlet), constant, gradient, and random value distributions defined on the same geometrical pattern.

To investigate the effects of image domain discretization and the amount of training data on the results of PINN model performance, nine sets of reference BVP images and corresponding FDM solutions were generated. These nine datasets are composed of 10, 40, and 70 thousand BVP images (further termed as 10k, 40k, and 70k datasets) including three different spatial resolutions (64 $\times$ 64, 128 $\times$ 128, 256 $\times$ 256), see Table 2. To enable the accurate performance of PINN models on arbitrary BVPs, a large variability in geometrical patterns and boundary conditions was considered by the generation of the reference set of BVP images. Accordingly, each set of images was generated by combining several strategies for the definition of the geometrical patterns (e.g., points, lines, contours, and solid shapes) and the spatial distribution of prescribed values (i.e. boundary conditions) including constant, gradient, and random distributions. In gradient distribution, the values for the pixels are assigned incrementally from 25 to 255 along different directions. Examples of BVP images for three different types of boundary conditions (including constant, gradient, and random distributions) are shown in Fig. 3. Furthermore, different directions and magnitudes of gradients were implemented to avoid potential biases in training data.

**Table 2:** Overview of nine image sets used for generation of reference FDM solutions and PINN model training.

Spatial resolution	70k images	40k images	10k images
64 $\times$ 64	Dataset 1	Dataset 2	Dataset 3
128 $\times$ 128	Dataset 4	Dataset 5	Dataset 6
256 $\times$ 256	Dataset 7	Dataset 8	Dataset 9

### Training and validation of forward PINN models

**Figure 4:**
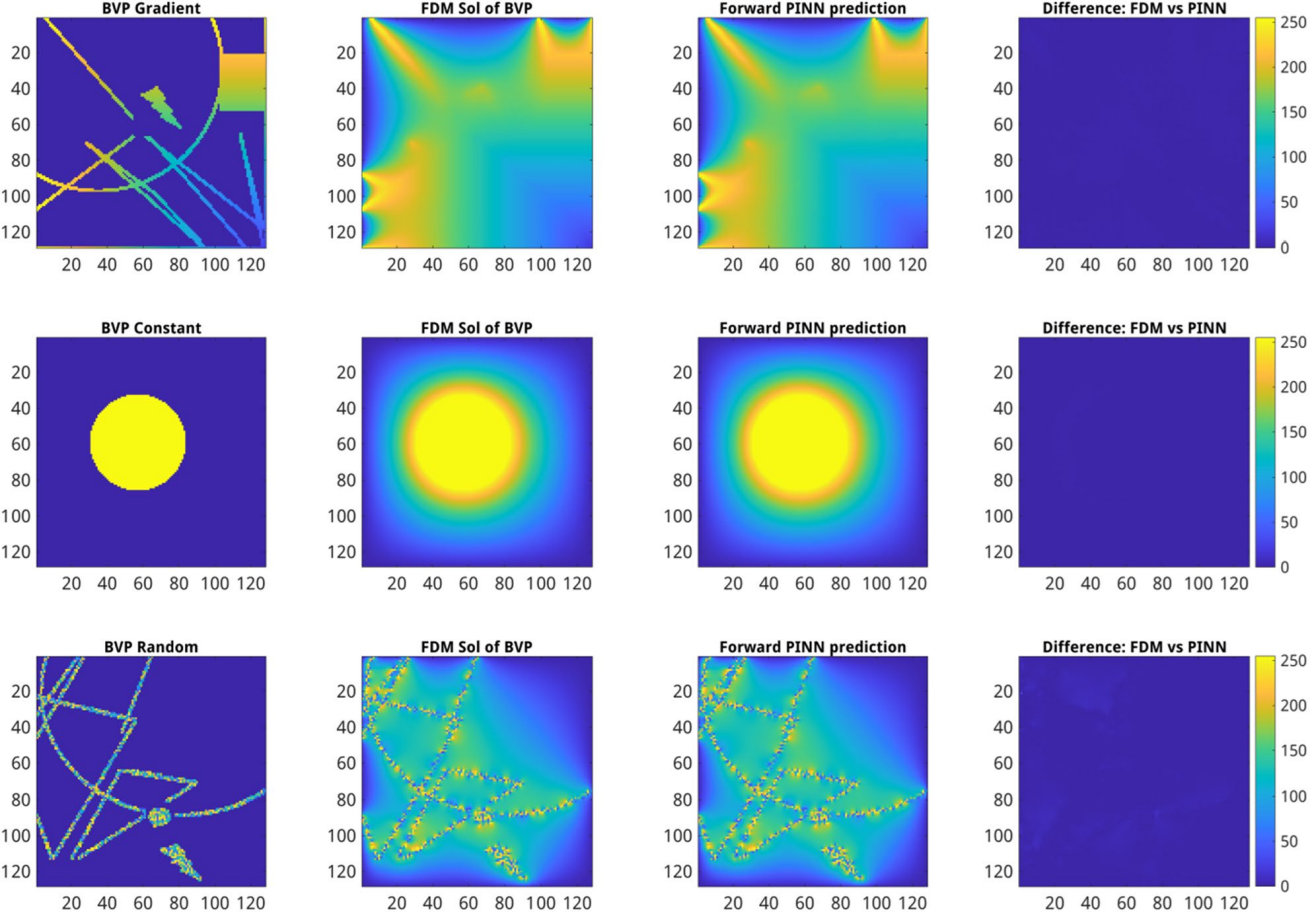
Exemplary comparison of FDM solutions vs. forward MSE U-Net model predictions trained on 10k 128 $\times$ 128 ground truth images. From left to right: (first column) original BVP images, (second column) FDM solutions of the 2D Laplace PDE calculated from BVP images, (third column) results of forward PINN model predictions computed from BVP images (first column), (fourth column) the absolute difference between FDM (second column) and forward PINN-predicted (third column) BVP images. From top to bottom: examples of gradient containing (top), constant (middle), and randomly distributed (bottom) boundary conditions. The color gradient indicates the intensity values and their difference ranging between [0, 255].

The forward PINN models were trained to emulate FDM solutions of 2D Laplace PDE using nine datasets from Table 2 as described in the “Methods” section. Examples of PINN model predictions based on the training dataset 4 from Table 2 including three different types of BVPs are shown in Fig. 4. Overall, PINN model predictions exhibit a high similarity with FDM solutions crossover all types of BVP images. However, a detailed analysis shows significant differences in PINN performance measures among different types of BVPs. One can see, for example, that the model performance is significantly more accurate when applied to BVP images with constant and gradient boundary conditions compared to randomly distributed boundary values in both methods, see Supplementary Fig. S1. In the case of the MC U-Net model, distinctive effects of spatial image resolutions on model training were observed. In particular, the MC U-Net model exhibited early overfitting within the first 50 epochs only when it was trained on 256 $\times$ 256 but not 64 $\times$ 64 and 128 $\times$ 128 BVP images. This can be traced back to the fact that the sample intervals in 256 $\times$ 256 FDM solutions are significantly smaller compared to 128 $\times$ 128 and 64 $\times$ 64 FDM solutions, which leads this model to capture more features in addition to the salient features. To address this issue, the maxpooling was increased from 2 to 4 instead which helped in mitigating the issue, see Supplementary Fig. S3. Thereby, the MSE U-Net based models trained with 10k datasets outperform the MC U-Net model trained with 70k datasets which is the best performing MC U-Net model see Supplementary Fig. S1. Additional performance measures for MC U-Net models can be found in Supplementary Materials Fig. S4–S6. The performance metrics of the forward models are presented in Table 3.

**Table 3:** Comparison of the performance of forward MC vs. MSE U-Net models trained on different datasets but evaluated on the same set of previously unseen BVP images.

Loss function	Training dataset	Accuracy (%)	F1 score (%)	MAE	MSE
SCE	Dataset 1	84.84	78.60	1.26	22.51
	Dataset 4	85.25	77.33	0.84	9.31
	Dataset 7	66.89	55.85	1.85	85.16
MSE	Dataset 3	83.26	77.61	1.49	2.36
	Dataset 6	93.66	91.18	0.49	0.88
	Dataset 9	90.61	83.97	0.69	3.2

### Training and validation of inverse solution models

**Figure 5:**
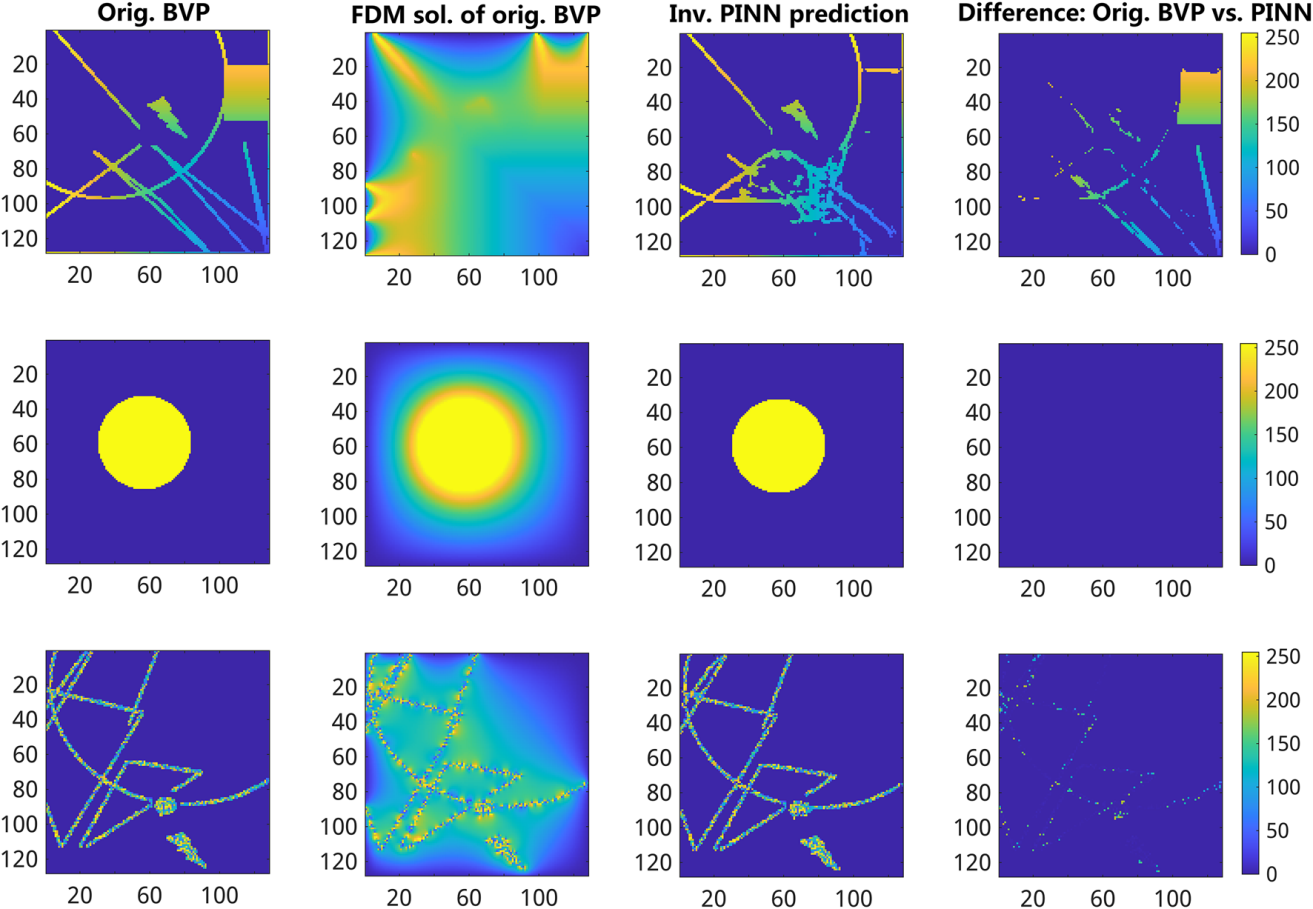
Exemplary comparison of original BVP images vs. inverse MSE U-Net model predictions trained on 10k 128 $\times$ 128 ground truth images. From left to right: (first column) original BVP images, (second column) FDM solutions of the 2D Laplace PDE calculated from BVP images, (third column) results of inverse PINN model predictions calculated from FDM solutions (second column), (fours column) the absolute difference between original (first column) and inversely PINN-predicted (third column) BVP images. From top to bottom: examples of gradient containing (top), constant (middle), and randomly distributed (bottom) boundary conditions. The color gradient indicates the intensity values and their difference ranging between [0, 255].

The inverse models are aimed to reconstruct the initial BVP (i.e. sparse image) from the solution of 2D Laplace PDE (i.e. smoothed images). Numerical solution of inverse problems using conventional methods is often a non-trivial task. Within the scope of PINN-based PDE solving, this task is trivially addressed by reversing the direction of the model training from target to source image sets, i.e. swapping input and output data used for training of forward models. Examples of the 128 $\times$ 128 inverse model performance for different types of BVP problems are shown in Fig. 5.

**Figure 6:**
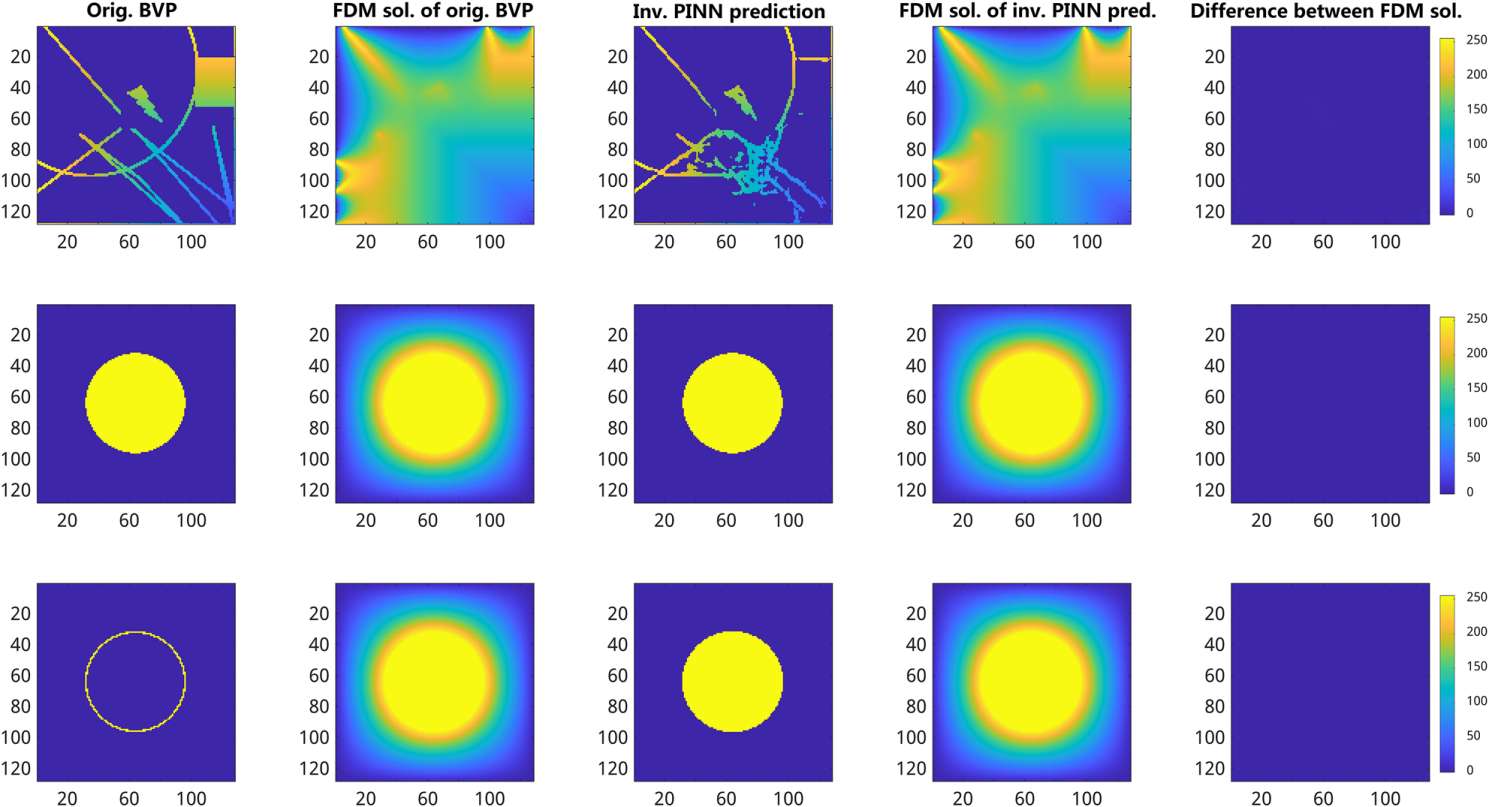
Validation of inverse PINN predictions vs. FDM. From left to right: (first column) original BVP image, (second column) FDM solution of 2D Laplace PDE for the boundary condition given by the original BVP image, (third column) reconstruction of the original BVP image from the FDM solution using the inverse PINN model, (fours column) FDM solution of the 2D Laplace PDE for the boundary conditions given by the inversely predicted BVP image, (fifth column) difference between FDM solutions (second and fours columns) computed from the original and inversely predicted BVP images (first and third columns). From top to bottom row: random BVP (top), solid (middle) vs. empty circle BVP (bottom) with the same boundary values. Despite differences between original and inversely reconstructed BVP images, their FDM solutions do not exhibit that large difference. In turn, the inverse PINN model fails to recover correct values inside the empty circle because of the principle ambiguity of inverse solutions of otherwise equal FDM images. The color map indicates intensity values and their differences in the range [0, 255]. The F1 scores between the FDM solutions of original BVP (second column) and the FDM solutions of inverse prediction (fourth column) are 99% (top row), 100% (middle row) and 100% (bottom row), respectively.

**Table 4:** Comparison of the performance metrics of inverse MC vs. MSE U-Net models trained on different datasets but evaluated using the same set of previously unseen BVP images.

Loss function	Dataset	Accuracy (%)	F1 score (%)	MAE	MSE
SCE	Dataset 1	94.16	82.48	6.89	1.2164e+03
	Dataset 3	90.66	71.81	10.89	1.8952e+03
MSE	Dataset 1	87.84	71.95	7.51	1.2030e+03
	Dataset 3	85.03	63.49	8.74	1.3921e+03

An exact recovery of original boundary conditions is, however, known to be associated with principle incompleteness, since different boundary conditions may sometimes lead to quite similar solutions. With both approaches, the predicted inverse boundary conditions are sparse compared to the original boundary conditions and the FDM computation for the sparsely predicted boundary conditions shows that the FDM solutions of inversely predicted boundary conditions show high similarity with the ground truth FDM solutions which proves the statement, see. Fig. 6. As one can see from the summary of the performance metrics for inverse U-Net models in Table 4, they exhibit a substantially larger MSE error than forward models cf. Table 3. It has been observed that the predicted boundary conditions are more sparse in the MSE U-Net models than in the MC U-Net models. Hence, MSE U-Net models exhibit lower F1 scores compared to MC U-Net models. Since the MSE U-Net models generate sparse inverse predictions, further analyses were done with the MC U-Net models see S2. Additional performance measures for MC U-Net models can be found in Supplementary Fig. S7–S9 and Supplementary Table S1.

**Figure 7:**
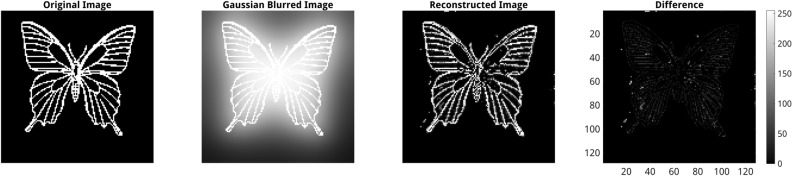
Example of application of the inverse 128 $\times$ 128 70k MC U-Net model for deblurring of a Gaussian smoothed image ($\sigma =8$, 12 iterations). From left to right: original image, Gaussian smoothed image, the result of deblurring of the Gaussian smoothed image using inverse PINN model. The color map indicates differences between the original and inversely reconstructed images in the range between [0, 255]. The F1 score between the original image and the reconstructed image is 67%.

From the view of image processing, the inverse 2D Laplace model effectively performs a kind of deblurring of smoothed images. Figure 7 shows an example of inverse reconstruction of the original image from its Gaussian smoothed version with $\sigma =8$ and a total of 12 iterations using the inverse 128 $\times$ 128 70k PINN model. Marginal differences between the original and inversely reconstructed images are due to differences between inverse PINN reconstruction (2D Laplacian) and image smoothing (2D Gaussian) algorithms. Another cause of the discrepancy of the inverse prediction from the original BVP image is due to principle ambiguity of inverse solutions, cf. Fig. 6.

### Comparison of computational PINN performance vs. FDM

**Figure 8:**
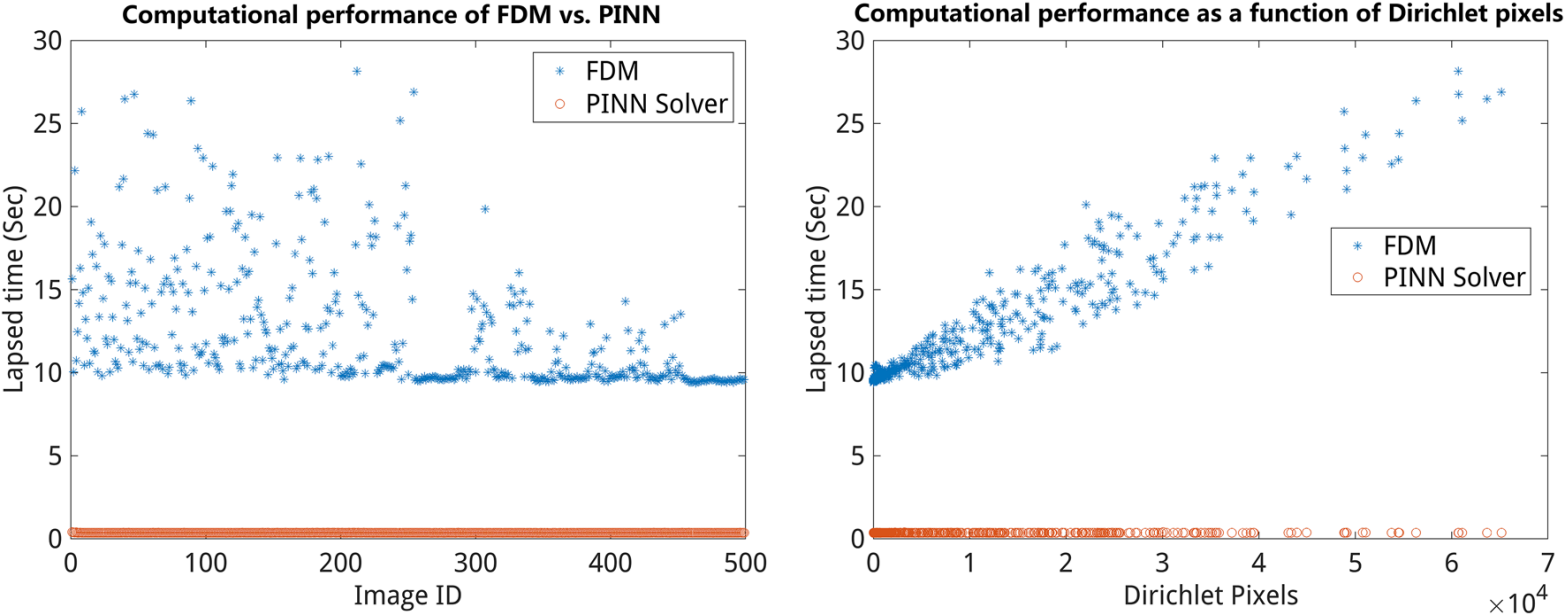
Comparison of computational PINN performance vs. FDM for 256 $\times$ 256 images. Left: lapsed time of PINN predictions (red) vs FDM solutions (blue) in seconds for 500 test BVP images. Right: lapsed time of PINN vs. FDM solutions depending on the number of pixels with prescribed values (Dirichlet pixels). The dependency of computational time on the number of Dirichlet pixels has to do with additional efforts for the implementation of prescribed boundary values.

Analysis of the computational performance of PINN models vs. FDM with 500 randomly selected BVP images among the 128 $\times$ 128 validation dataset which contains examples of all possible BVPs. The average time taken by the PINN solver is compared against the FDM solution in dependency on image resolution as well as the number of nodes with prescribed boundary condition (i.e. Dirichlet pixels) as it determines sparseness of the stiffness matrix and its numerical solution using PCG. A summary of the computational performance of pre-trained PINN models vs. FDM is shown in Table 5 and Fig. 8. As one can see computation of solutions of 2D Laplace PDE using PINN models is much more efficient in comparison to the iterative solutions using FDM. Even if one considers the computational time of FDM varies on the number of Dirichlet pixels for the same image size, it does not have a large effect. With increasing image resolution computation performance of FDM grows significantly faster compared to PINN models that perform the computation of images up to 256 $\times$ 256 size in less than 1 s.

**Table 5:** Summary of computational performance for forward PINN models vs. FDM solutions computed for a set of 500 unseen BVP images.

Image size	Average number of Dirichlet pixels	Average FDM performance (s)	Average PINN performance (s)
64 $\times$ 64	788	0.054	0.05
128 $\times$ 128	2579	0.70	0.19
256 $\times$ 256	10172	12	0.37

### Implementation of 2D Laplace PINN solver as a command line demo tool

Our 2D Laplace PINN solver was implemented as an executable demo tool that can be run on Windows and Linux OS using a simple command line call: model.exe <input.png> <output.png> <model.h5>. It can be downloaded along with example images from https://ag-ba.ipk-gatersleben.de/Lap2Dpinn.html.

## Discussion

In this study, we developed and investigated two PINN models for solving arbitrary 2D Laplace boundary value problems defined on 8-bit images. Our experimental results showed that PINN models trained on a large set of reference FDM solutions are capable of realistically predicting solutions of new, previously unseen forward as well as inverse 2D Laplace BVPs with remarkably high accuracy but in a much shorter computational time compared to FDM.

To cover a large range of boundary value problems, PINN models were trained on different types of boundary value problems. While all these types of BVPs are mathematically admissible, some of them appear to be more physically meaningful (constant or gradient boundary conditions) than others (randomly distributed boundary values). From this viewpoint, it is not surprising that the accuracy in the prediction of physically more meaningful BVPs is higher than in the case of random boundary conditions. In general, the choice of suitable types of BVPs depends on the concrete physical problem, but in the case of 2D Laplace PDE, random boundary conditions appear to be not physically relevant.

Both forward and inverse 2D Laplace BVPs turn out to be solvable using PINN models in a non-iterative manner with remarkable accuracy. However, comparing the performance of forward vs. inverse PINN models shows that forward models make more accurate predictions than inverse models. This is, however, not surprising as different boundary conditions can produce similar solutions. Two alternative loss functions were used for PINN model training. In the case of forward PINN models, the U-Net models trained with the MSE loss function show slightly better performance compared to the multiclass U-Net models that were trained with the SCE loss functions. This was, however, not observed for inverse predictions, which can be attributed to principle ambiguity in the inverse solutions and larger errors compared to forward solutions^47^. However, due to the restriction of the SCE measure to integer-valued classes, the MSE loss function has a more general spectrum of applications. Evaluation of PINN model predictions was done using several different metrics. However, as already known from previous works, quantification of accuracy using the F1 score measure is more advantageous, especially in the case of uneven distributions such as, in particular, the case of inverse problems with sparse boundary conditions. The PINN models developed in this work take raw input images and do not require any further efforts for domain discretization, assembly of the linear system of equations, and its iterative solution. These features make them an efficient and easy-to-use tool for straightforward application, especially, for cross-disciplinary problems. However, there is still certain technical (i.e. GPU) limitations concerning the size of image domains for which PINN models can be trained. Even if these technical burdens can be overcome in the future, further investigation of PINN solving techniques relying on unstructured point clouds^48^ appears to be of general interest.

The current work represents a feasibility study with application to the 2D Laplace problem defined on the integer-valued image domain. Extensions of the PINN approach to a more general class of floating point valued and/or multidimensional BVPs using a more general MSE loss function are straightforward. Further investigations are required to dissect the principle capabilities and accuracy bounds of the suggested PINN approaches by application to other image and physics-based boundary value problems.

## Supplementary Information

Supplementary Information.

## Data Availability

Supplementary Information with example images accompanies this manuscript. Further datasets used and analyzed during the current study are available from the corresponding author upon reasonable request.
