# Supplementary material for: FDM data driven U-Net as a 2D Laplace PINN solver
Source: Sci Rep. 2023 Jun 5;13:9116. doi: 10.1038/s41598-023-35531-8 (PMC10241951; doi:10.1038/s41598-023-35531-8)
Supplement: Supplementary file 1 — Supplementary Information. [file 41598_2023_35531_MOESM1_ESM.pdf]

Supplementary Material

FDM data driven U-Net as a 2D Laplace PINN solver

Anto Nivin Maria Antony, Narendra Narisetti, and Evgeny Gladilin

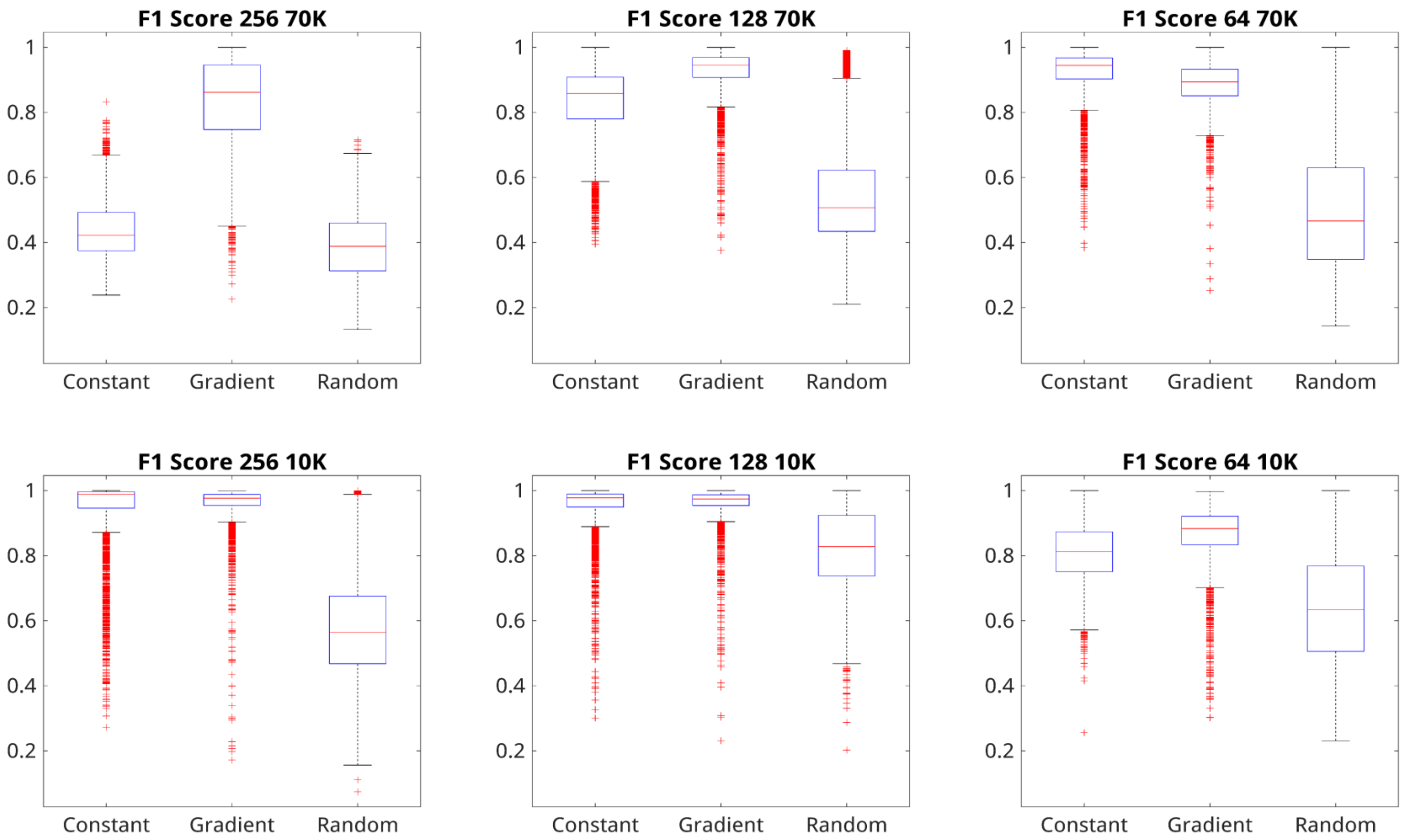

Figure S1: Overview of the forward PINN model performance in terms of F1 scores: (upper row) MC U-Net models, (bottom row) MSE U-Net models.

Interpretation of boxplots: The upper limit of the black line in each box plot indicate the maximum value, the lower limit indicates the minimum value, the upper limit and the lower limit of the blue box indicate the 75th percentile and the 25th percentile respectively and the red line in the middle of the box plots indicates the median value. Higher the value of F1 score better is the prediction.

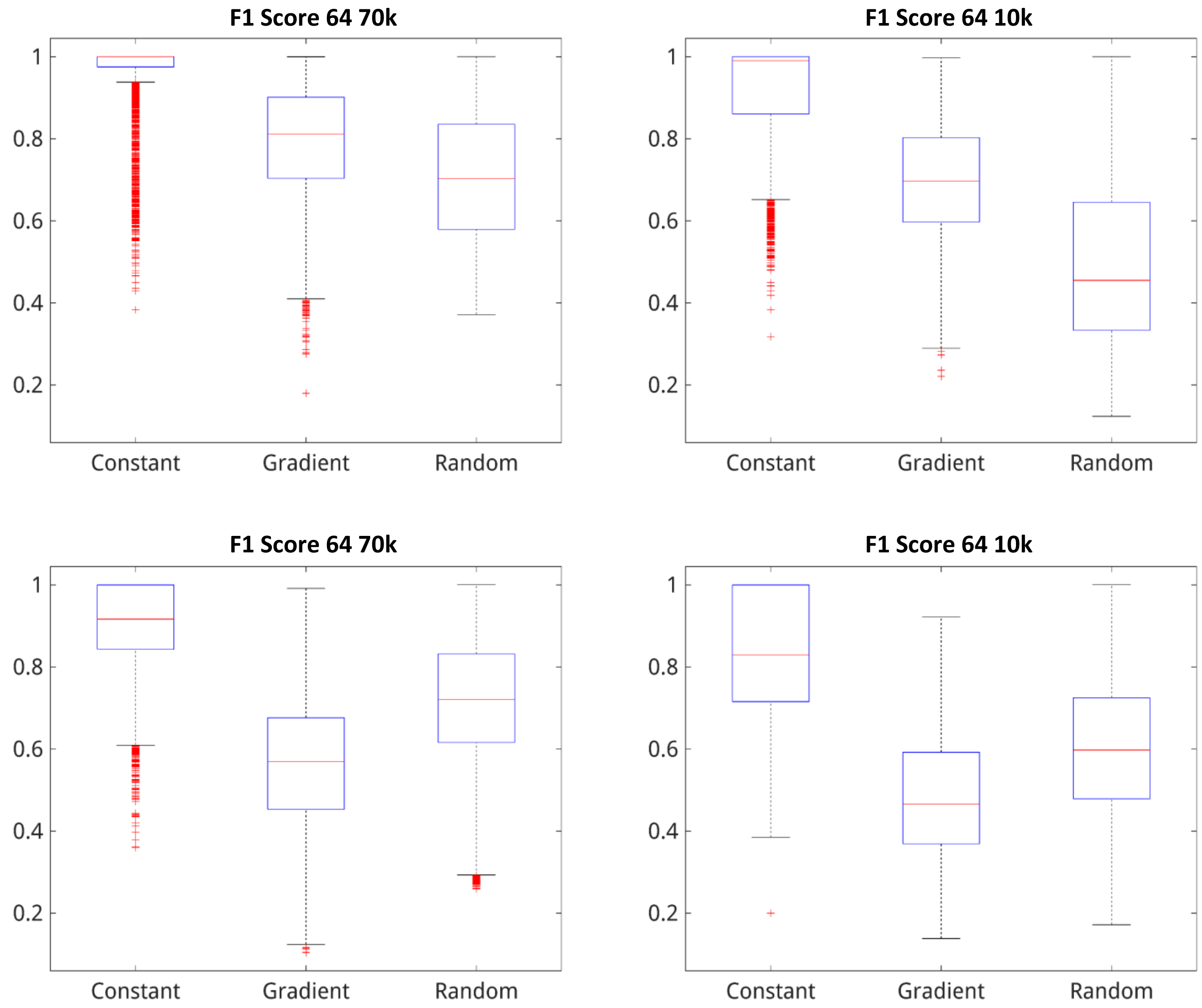

Figure S2: Overview of the inverse PINN model performance in terms of F1 scores: (upper row) MC U-Net models, (bottom row) MSE U-net models.

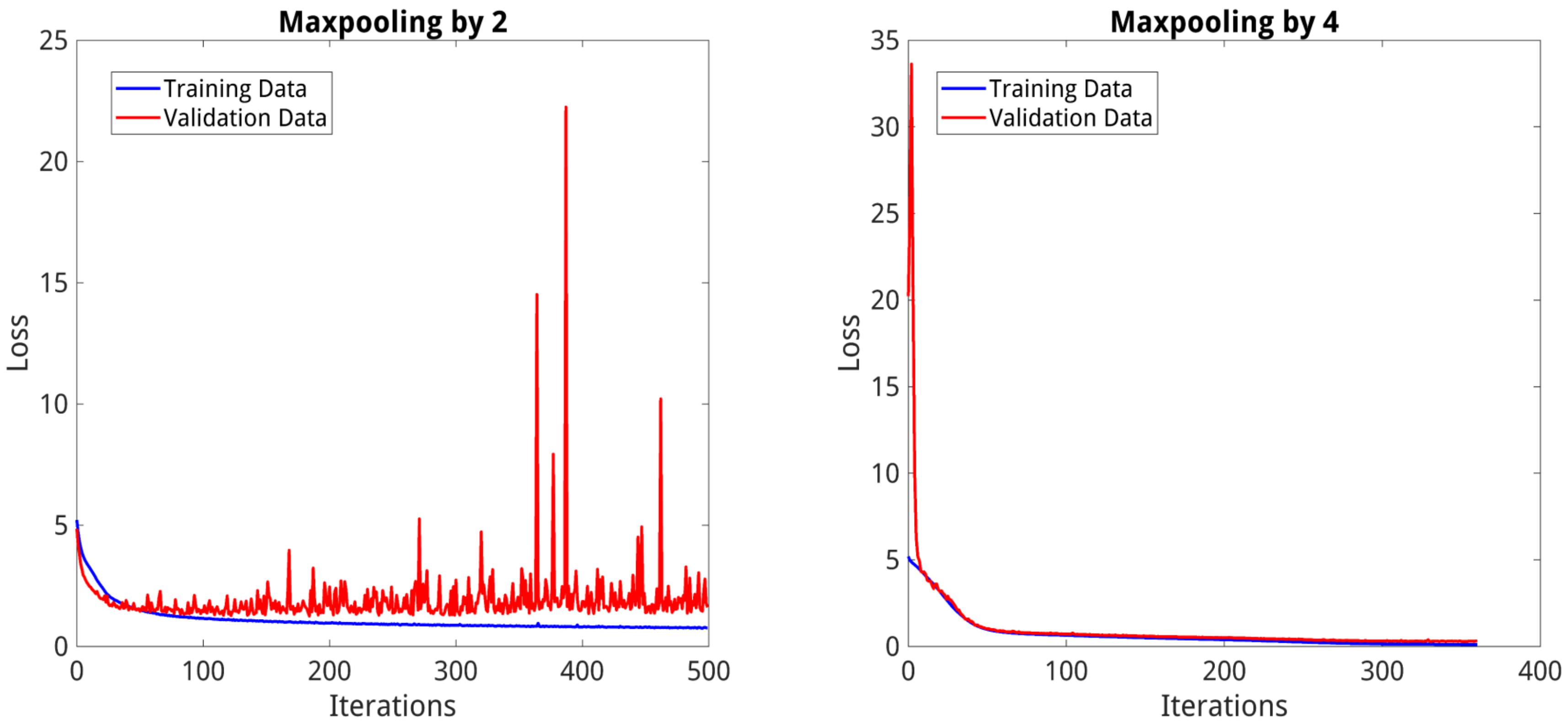

Figure S3: Dealing with overfitting of the 256x256 MC U-net model. From left to right: (left) the 256x256 MC U-net model with maxpool operation by 2 exhibits a pattern of overfitting in course of iterative training, i.e. divergence of performance between training and testing as well as large oscillations in accuracy (loss) between subsequent training iterations; (right) the model trained with maxpool operation by 4 shows no such overfitting.

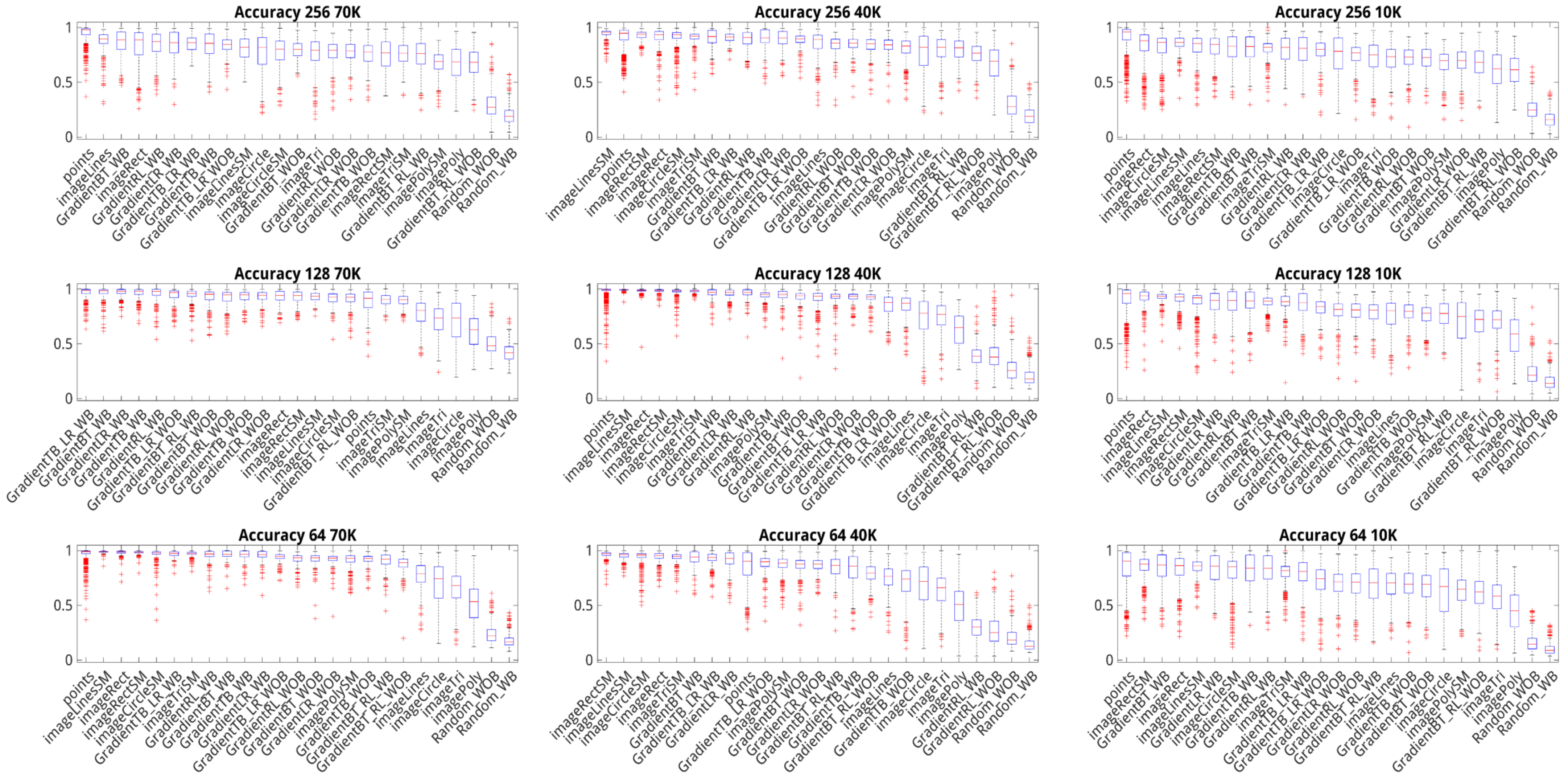

Figure S4: Summary of accuracy of nine forward MC U-Net models from Table 1 for different types of BVPs including the sub-categories of constant, gradient and random boundary conditions.

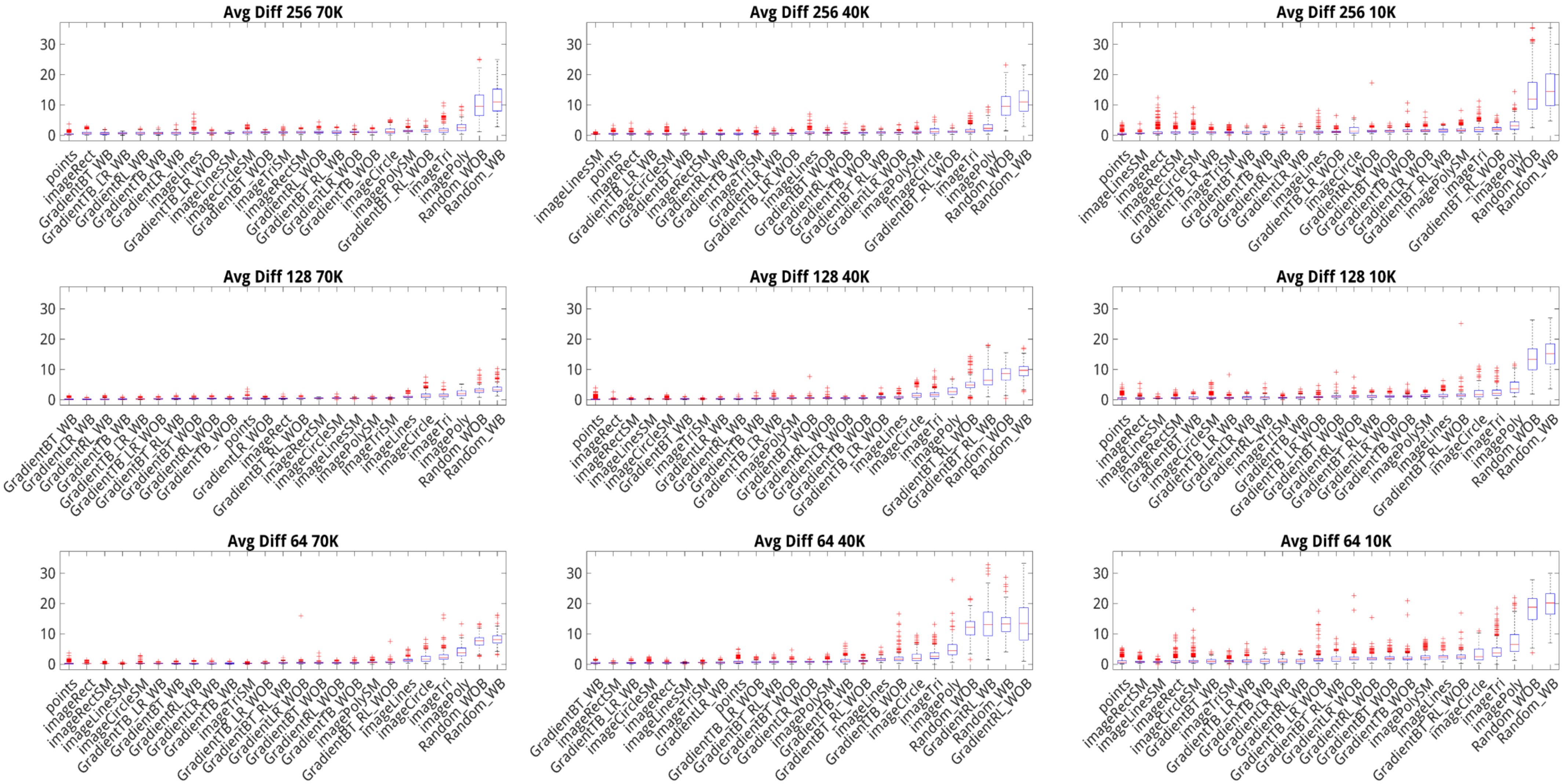

Figure S5: Summary of average difference of nine forward MC U-Net models from Table 1 for different types of BVPs including the sub-categories of constant, gradient and random boundary conditions.

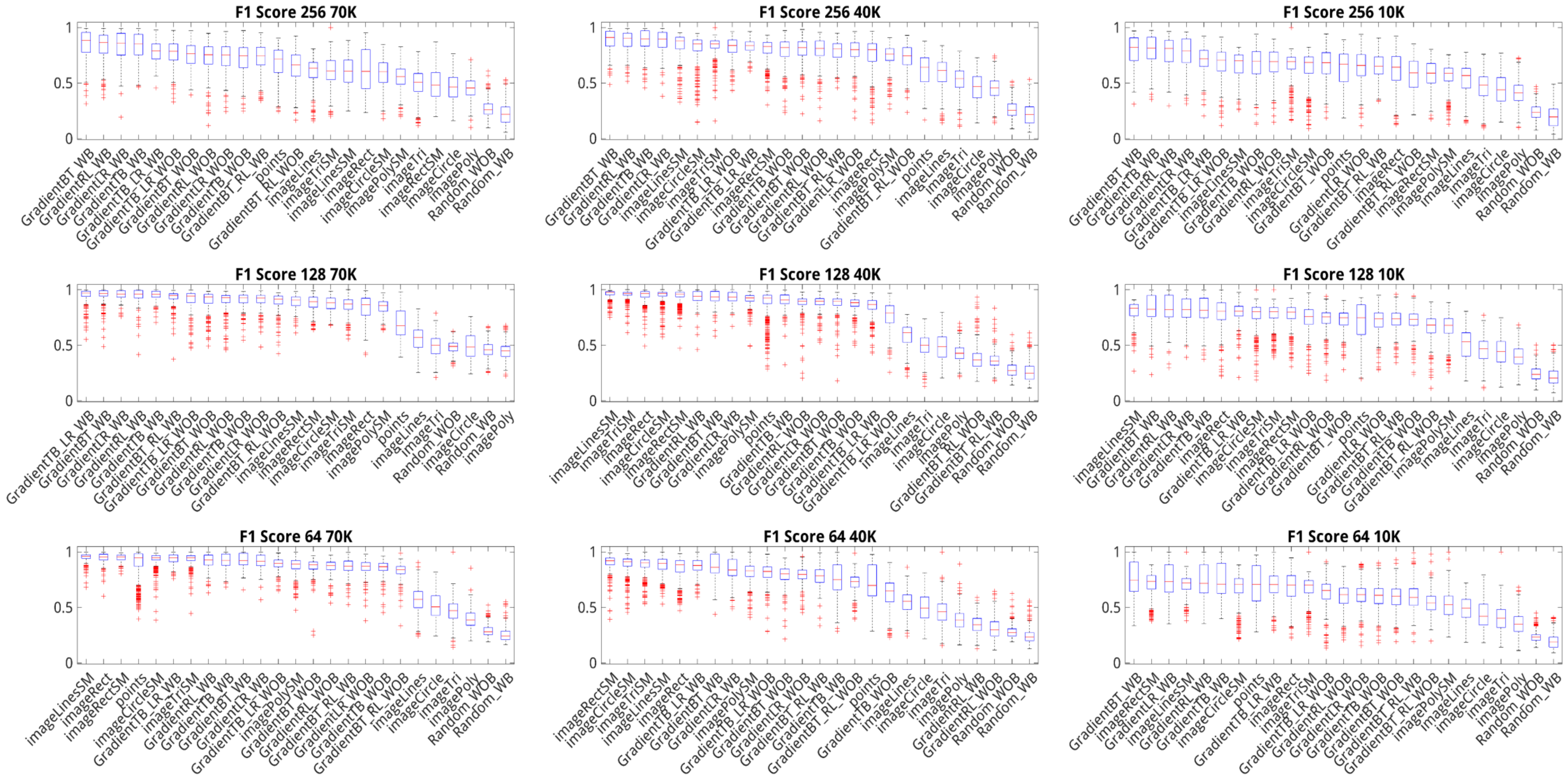

Figure S6: Summary of F1 Score of nine forward MC U-Net models from Table 1 for different types of BVPs including the sub-categories of constant, gradient and random boundary conditions.

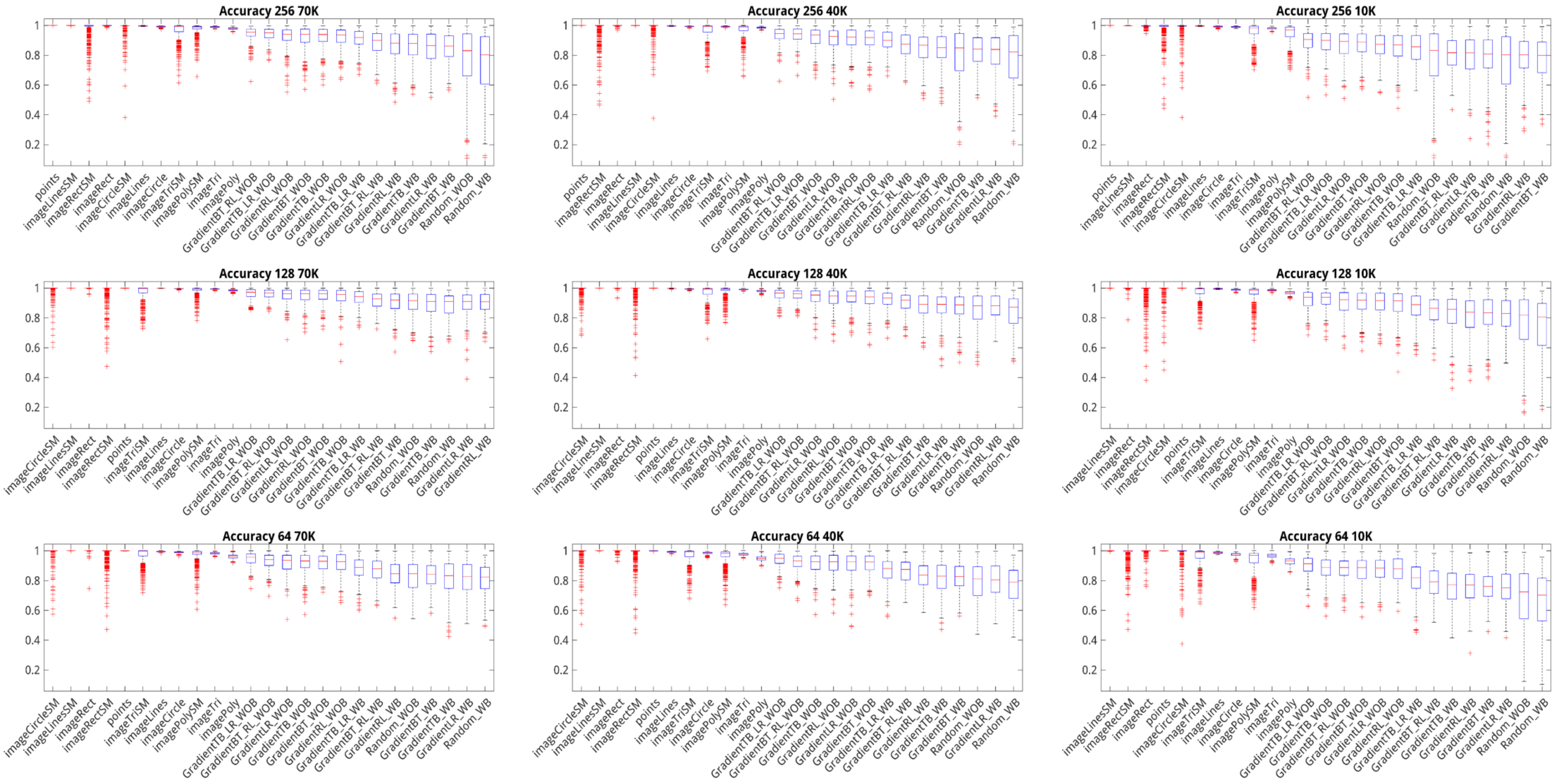

Figure S7: Summary of accuracy of nine inverse MC U-Net models from Table 1 for different types of BVPs including the sub-categories of constant, gradient and random boundary conditions.

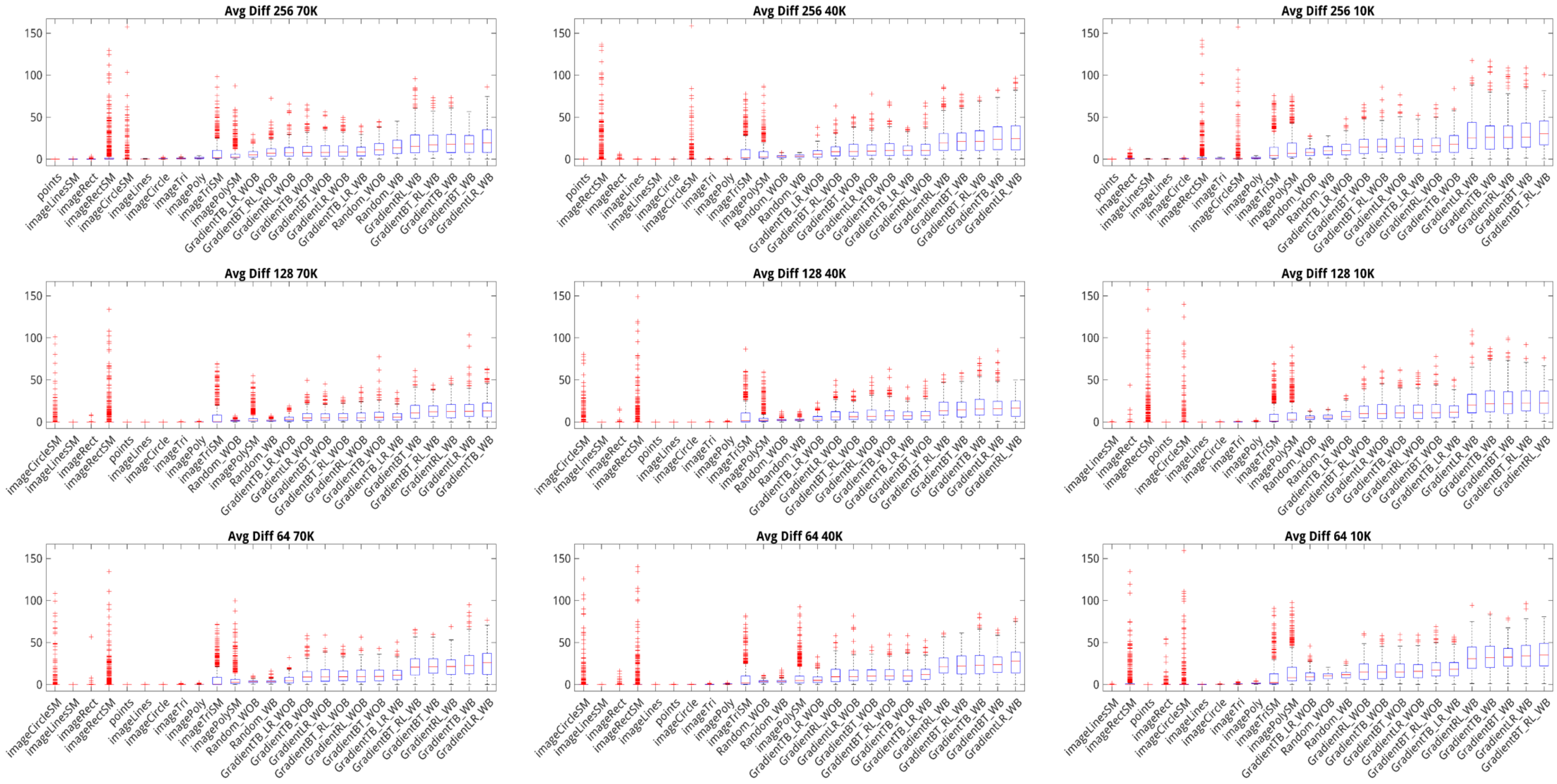

Figure S8: Summary of average difference of nine MC U-Net from Table 1 for different types of BVPs including the sub-categories of constant, gradient and random boundary conditions.

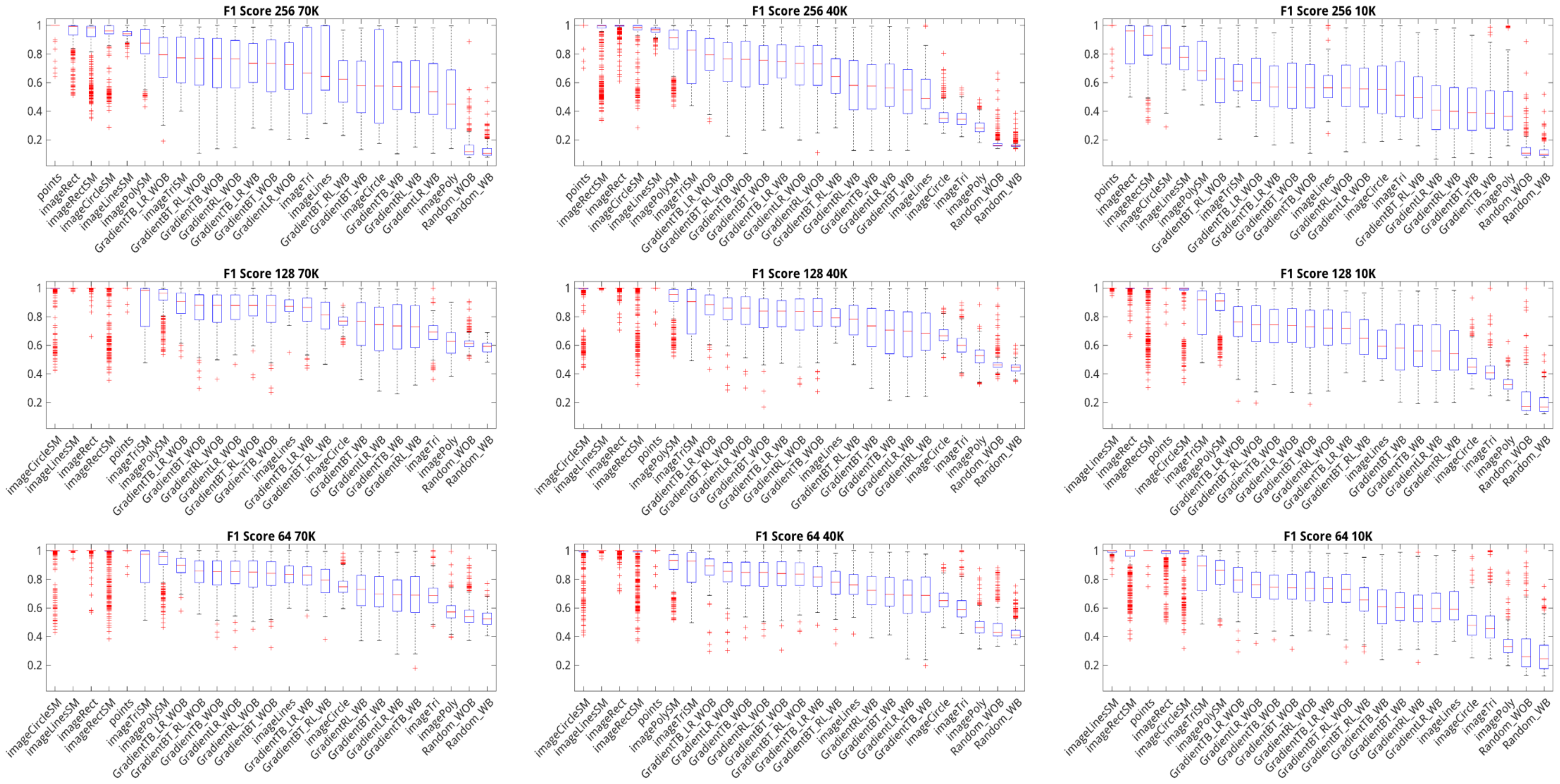

Figure S9: Summary of F1 Score of nine inverse MC U-Net from Table 1 for different types of BVPs including the sub-categories of constant, gradient and random boundary conditions.

Indicators	Explanation
*_WB	With Borders
*_WOB	Without Borders
*_RL	Gradient applied from right to left (-180°)
*_LR	Gradient applied from left to right (180°)
*_BT	Gradient applied from Bottom to Top (90°)
*_TB	Gradient applied from Top to Bottom (-90°)

Table S1: Summary of abbreviations used in naming the BVPs and their corresponding explanations. For example, Gradient_TB_LR_WOB – gradient increases from the left top image corner (1,1) towards the right bottom image corner (N,N) at an angle 225.
